# Minimal sufficient balance randomization for sequential randomized controlled trial designs: results from the ESCAPE trial

**DOI:** 10.1186/s13063-017-2264-1

**Published:** 2017-11-02

**Authors:** Tolulope T. Sajobi, Gurbakhshash Singh, Mark W. Lowerison, Jordan Engbers, Bijoy K. Menon, Andrew M. Demchuk, Mayank Goyal, Michael D. Hill

**Affiliations:** 1 Department of Community Health Sciences and O’Brien Institute for Public Health, Cumming School of Medicine, University of Calgary, Calgary, AB Canada; 2 Department of Clinical Neurosciences, Cumming School of Medicine, University of Calgary, 3300 Hospital Drive NW, Calgary, AB T2N 4N1 Canada; 3 Clinical Research Unit, Cumming School of Medicine, University of Calgary, Calgary, AB Canada; 4 Hotchkiss Brain Institute, Cumming School of Medicine, University of Calgary, Calgary, AB Canada; 5 Department of Radiology, Cumming School of Medicine, University of Calgary, Calgary, AB Canada

**Keywords:** Minimal sufficient balance, Randomization, Stroke trial, Endovascular therapy, Data monitoring

## Abstract

**Background:**

We describe the implementation of minimal sufficient balance randomization, a covariate-adaptive randomization technique, used for the “Endovascular treatment for Small Core and Anterior circulation Proximal occlusion with Emphasis on minimizing CT to recanalization times” (ESCAPE) trial.

**Methods:**

The ESCAPE trial is a prospective, multicenter, randomized clinical trial that enrolled subjects with the following main inclusion criteria: less than 12 h from symptom onset, age 18 years or older, baseline NIHSS score > 5, ASPECTS score > 5 and computed tomography angiography (CTA) evidence of carotid T/L or M1-segment middle cerebral artery (MCA) occlusion, and at least moderate collaterals by CTA. Patients were randomized using a real-time, dynamic, Internet-based, minimal sufficient balance randomization method that balanced the study arms with respect to baseline covariates including age, sex, baseline NIHSS score, site of arterial occlusion, baseline ASPECTS score and treatment with intravenously administered alteplase.

**Results:**

Permutation-based tests of group differences confirmed group balance across several baseline covariates including sex (*p* = 1.00), baseline NIHSS score (*p* = 0.95), site of arterial occlusion (*p* = 1.00), baseline ASPECTS score (*p* = 0.28), treatment with intravenously administered alteplase (*p* = 0.31), and age (*p* = 0.67).

**Conclusion:**

Results from the ESCAPE trial demonstrate the feasibility and the benefit of this covariate adaptive randomization scheme in small-sample trials and for data monitoring endeavors.

**Trial registration:**

ESCAPE trial – NCT01778335 – at www.clinicaltrials.gov. Registered on 29 January 2013.

**Electronic supplementary material:**

The online version of this article (doi:10.1186/s13063-017-2264-1) contains supplementary material, which is available to authorized users.

## Background

Stratified randomization is commonly used to achieve balance in randomized controlled trials (RCTs) but may not always lead to balanced treatment groups, especially when there are multiple baseline covariates that are relevant prognostic factors [1–4]. Treatment group imbalance across baseline covariates could lead to erroneous estimation and interpretation of treatment effect sizes reported in RCTs both at interim and final analyses [5]. Although primary analyses that adjust for imbalance in baseline covariates remains a popular method for dealing with baseline covariate imbalance [6–8], ignoring imbalance across important baseline prognostic factors during randomization may result in significant loss in statistical power to detect treatment differences, even if these factors are adjusted for in analysis. In addition, balance at interim analysis, when sample sizes are necessarily small, is important to permit confident decision-making by the Data Safety and Monitoring Committee. As a result of increased awareness of the potential for loss of power, balancing treatment allocation for influential covariates has become more important in modern clinical trials [9].

Covariate adaptive randomization designs have been proposed as an alternative to stratified randomization. This class of randomization design is advantageous in that it is able to achieve between-group balance across baseline covariates even in small-sample-size RCTs [10–14]. Covariate adaptive randomization designs are particularly useful in stroke trials where treatment groups may vary on multiple but influential baseline covariates; these include treatment with intravenously administered alteplase and baseline stroke severity among others. Commonly used adaptive randomization strategies to balance baseline covariates include stratified constrained randomization and minimization [15, 16]. However, both strategies can only balance on few categorical baseline covariates and may lead to imbalance especially in continuous, mixed continuous/discrete covariates [17]. Minimal sufficient balance randomization is a recent, more robust covariate adaptive randomization strategy for use in RCTs with several multiple baseline covariates [18, 19]. This technique is efficient in sequential RCT designs where baseline covariates are expected to be balanced at each interim monitoring.

In this study, we describe the implementation of minimal sufficient balance randomization for the “Endovascular treatment for Small Core and Anterior circulation Proximal occlusion with Emphasis on minimizing CT to recanalization times” (ESCAPE) trial, a multicenter RCT that enrolled 316 acute-ischemic-stroke subjects [20, 21]. We provide recommendations about the potential use of minimal sufficient balance for sequential designs in stroke trials.

## Methods

### Minimimum sufficient balance randomization

Consider *K* baseline covariates to be balanced in the randomization process between two treatment arms in a trial. To allocate a subject for randomization, the distributions of each baseline covariate between the two treatment arms are evaluated based on the *p* values of the imbalance tests (i.e., *t* test for continuous and chi-squared test for categorical covariates). For example, for the *k*th (*k* = 1, 2….*K*) covariate, let *n*_*A*_ and *n*_*B*_ be the total number of subjects previously randomized to treatment arms *A* and *B*, respectively. Assume that ${\overline{x}}_{ka}$, ${s}_{ka}\kern0.24em {\overline{x}}_{kb}$, and *s*_*kb*_ are the mean and standard deviation (SD) for treatment arms A and B, respectively, on the *k*th covariate. Let *t*_*k*_ *p*_*k*_, *t*_k_^*^, and *p*_k_^*^ represent the *k*th variable’s test statistic, the corresponding *p* value, 97.5th quantile of the *t* distribution, and control limit, respectively. For the current subject to be randomized, the choice between a biased coin and simple random assignment will be based on the test results and the current subject’s baseline covariate value *x*_*k*_:

1 $$ {\displaystyle \begin{array}{l} Vote for\ A\kern1.75em if\ \left[\left({t}_k<-{t}_k^{\ast}\right)\kern0.75em and\kern0.5em \left({x}_k>{\overline{x}}_{xb}\right)\right]\kern0.5em or\kern0.5em \left[\left({t}_k>{t}_k^{\ast}\right)\kern0.5em and\kern0.5em \left({x}_k<{\overline{x}}_{xb}\right)\right]\\ {} Vote for\ B\kern1.75em if\ \left[\left({t}_k<-{t}_k^{\ast}\right)\kern0.75em and\kern0.5em \left({x}_k>{\overline{x}}_{xa}\right)\right]\kern0.5em or\kern0.5em \left[\left({t}_k>{t}_k^{\ast}\right)\kern0.5em and\kern0.5em \left({x}_k<{\overline{x}}_{xa}\right)\right]\\ {} Neutral\kern2.7em if\kern0.5em otherwise\end{array}}, $$

where

$$ {t}_k=\frac{\sqrt{n_A{n}_B}\left({\overline{X}}_{kA}-{\overline{X}}_{kB}\right)}{\sqrt{n_B{s}_{kA}^2+{n}_A{s}_{kB}^2}}. $$

Based on the above rule (1), if the current subject has a covariate value between the two treatment groups’ average values, neither treatment is favored because the two possible allocations, treatment arm *A* or *B*, for the current subject yield little difference in the imbalance of the covariate. On the other hand, for the *k*th categorical baseline covariate with *g* categories, let *n*_*kjA*_ and *n*_*kjB*_ represent the observed number of subjects in the category *j* (*j* = 1…g) of covariate *k* previously randomized to treatment arms A and B, respectively. Let *E*_*kjA*_ and *E*_*kjB*_ be the expected number of subjects in the *j* category being allocated to treatment arms A and B, respectively. Then:

$$ {\chi}_k^2=\sum \limits_{j=1h=A,B}^g\sum \kern1em \frac{{\left({n}_{kjh}-{E}_{kjh}\right)}^2}{E_{kjh}}, $$

where

$$ {E}_{kjh}=\frac{\left({n}_{kjA}+\kern0.5em {n}_{kjB}\right)\sum \limits_{j=1}^g{n}_{kjh}}{\sum \limits_{j=1}^g\left({n}_{kjA}+\kern0.5em {n}_{kjB}\right)}. $$

Based on the chi-squared distribution with *g* – 1 degrees of freedom, if *p*_*k*_ < *p*_k_^*^, then:

$$ {\displaystyle \begin{array}{l} Vote for\ A\kern1.75em if\ \left({p}_k<{p}_k^{\ast}\right)\kern0.5em and\kern0.75em \left({E}_{kjA}>{n}_{kjA}\right)\\ {} Vote for\ B\kern1.75em if\ \left({p}_k<{p}_k^{\ast}\right)\kern0.5em and\kern0.75em \left({E}_{kjB}>{n}_{kjB}\right)\\ {} Neutral\kern2.8em if\kern0.5em otherwise\end{array}}. $$

After dealing with imbalances among baseline covariates, the probability for assigning the current subject to treatment *A* is described as:

$$ \Pr \left\{\mathrm{Assign}\kern0.5em \mathrm{current}\kern0.5em \mathrm{patient}\kern0.5em \mathrm{to}\kern0.5em A\right\}\kern0.5em =\kern0.5em \left\{\begin{array}{l}\upxi \kern3.5em \mathrm{if}\kern0.5em \mathrm{Treatment}\kern0.5em A\kern0.5em \mathrm{received}\kern0.5em \mathrm{more}\kern0.5em \mathrm{votes}\\ {}1\kern0.5em -\upxi, \kern1.5em \mathrm{if}\kern0.5em \mathrm{Treatment}\kern0.5em B\kern0.5em \mathrm{received}\kern0.5em \mathrm{more}\kern0.5em \mathrm{votes}\kern0.5em \\ {}0.5\kern2.7em \mathrm{otherwise}.\end{array}\right., $$

where *ε* is the biased coin probability. A probability range of 0.60 ≤ *ξ* ≤ 0.70 has been recommended for a balanced two-arm trial [18].

### Minimal sufficient balance in the ESCAPE trial

The ESCAPE trial was a randomized controlled trial, with open-label treatment and blinded outcome evaluation that used a superiority design, and was conducted in Canada, US, Europe, UK, and South Korea. It tested whether endovascular treatment was superior to best medical care (including intravenous thrombolysis with alteplase) for subjects with major acute ischemic stroke due to proximal large-vessel occlusion proven with computed tomography (CT) imaging. The primary outcome was the 90-day functional outcome score measured on the modified Rankin Scale; this is a standard, well-accepted outcome for acute stroke treatment trials. The trial sample size was planned for *N* = 500 and the predicted effect size was a common odds ratio of 1.8 (see Additional file 1). All subjects or their surrogate provided written informed consent and the study protocol was approved at each site by the Local Ethics Board or equivalent.

The minimal sufficient balance randomization process is implemented using a server-based system. The distribution balance will be achieved on six baseline covariates: age, sex, baseline stroke severity (measured by the National Institutes of Health Stroke Severity Scale (NIHSS) score), site of arterial occlusion, baseline Alberta Stroke Program Early CT score (ASPECTS) score, and treatment with intravenously administered alteplase (Table 1). Because randomization occurred dynamically in real time, treatment allocation was fully concealed. At the point of randomization, six baseline covariates were entered into an on-line data system. The system depends upon the real-time accuracy (or near accuracy) of these variables. Variables that are not known or cannot be known in real time cannot be used for this process.

**Table 1:** Pairwise correlation (Pearson’s rho) among the six baseline covariates used in the minimal sufficient balance randomization procedure

	Age (years)	Sex	NIHSS score	ASPECTS score	Occlusion location	Alteplase (tPA)
	Continuous variable	Binary variable (male/female)	Continuous integer variable (score from 6 to 42)	Binary variable (good score 8–10 vs. moderate score 6–7)	Binary variable (MCA vs. ICA)	Binary variable (true/false)
Age	1.000					
Sex	0.1587	1.000				
NIHSS score	0.2125	−0.0141	1.000			
ASPECTS score	0.1125	0.0330	−0.1474	1.000		
Occlusion location	0.0660	0.0144	− 0.2398	0.0276	1.000	
Alteplase (tPA)	− 0.0394	− 0.0610	0.0022	0.0274	− 0.0133	1.000

*NIHSS* National Institutes of Health Stroke Severity Scale, *ASPECTS* Alberta Stroke Program Early CT score, *tPA* tissue plasminogen activator, *MCA* middle cerebral artery, *ICA* internal carotid artery

These six covariates were chosen a priori because they are known key prognostic variables after acute stroke

In the ESCAPE trial, data on the first 40 subjects (20 in each treatment arm) were allowed to accrue (i.e., burn-in) before the randomization algorithm was allowed to kick in. The probability of biased coin was set at 0.60, while the control limits were set at *p*_k_^*^ = 0.10 for each covariate. A stratified minimal sufficient balance randomization was used in this trial with respect to intravenously administered alteplase (tPA) treatment. Specifically, minimal sufficient balance randomization was used to dynamically allocate subjects to treatment or control arms for subjects who did or did not receive intravenously administered alteplase while balancing on the remaining five covariates. Overall, the six covariates considered in the ESCAPE trial were entered into the algorithm as recorded by the site enrolling physicians. This set of variables was maintained in the randomization database. If one of those six variables later changed value (due to new information becoming available), the trial database was considered the source of truth and that specific subject variable was updated in the randomization database. In this way, the distribution of covariates was continually updated as the trial progressed. Consistent with previous research recommendations about covariate-adaptive randomization schemes [8, 15, 22], a permutation test was used to assess the evidence of overall balance among treatment arms at interim analysis and the end of the trial.

## Results

At the interim analysis of the actual data (*N* = 243), 183 subjects received intravenously administered alteplase, while 59 subjects did not receive it. There were no significant differences between both arms of the trial on the baseline covariates in subjects who received intravenously administered alteplase and those who did not receive it, suggesting an overall balance in minimal sufficient balance patient allocation into both treatment arms (see Table 2). In the group that did not receive intravenously administered alteplase, the proportion of subjects that had true random assignment (after burn-in) was 87.2%, while the minimal sufficient balance algorithm exhibited true randomness (i.e., there was no voting on any of the balancing covariate) in dynamically allocating the last 30 subjects. On the other hand, 97.5% of the subjects who received the intravenously administered alteplase were truly randomly assigned (after burn-in) and there was no voting on any of the balancing covariate for the last 66 subjects randomized before interim analysis.

**Table 2:** Treatment differences on baseline covariates by intravenously administered alteplase status at interim analysis (*N* = 243)

Baseline covariates	IV alteplase (*n* = 183)			Without IV alteplase (*n* = 59)		
	Treatment (*n* = 96)	Control (*n* = 87)	*p* value	Treatment (*n* = 36)	Control (*n* = 23)	*p* value
Age (mean, SD)	68.82 (12.24)	68.69 (14.78)	0.95	73.56 (13.92)	70.26 (14.97)	0.40
NIHSS score (mean, SD)	16.54 (5.41)	17.28 (5.75)	0.38	17.83 (5.81)	15.78 (5.21)	0.16
Sex (% female)	47.92%	51.72%	0.61	52.78%	56.52%	0.78
ASPECTS score (% (8–10))	68.75%	68.97%	0.98	66.67%	73.91%	0.56
Baseline occlusion location (% MCA M1)	70.83%	70.11%	0.92	72.22%	73.91%	0.89

*ASPECTS* Alberta Stroke Program Early CT score, *NIHSS* National Institute of Health Stroke Severity Scale, *MCA M1* M1 segment of middle cerebral artery, *SD* standard deviation, IV intravenously administered

At the end of the trial, 237 subjects received the intravenously administered alteplase, while 78 subjects did not receive it. A permutation test of overall group differences revealed no evidence of imbalance on each baseline covariate when the trial was stopped for overwhelming efficacy (see Table 3). In the group that did not receive intravenously administered alteplase, 91.4% of the subjects were truly randomly assigned (after burn-in) at the end of the trial, while there was no voting on any of the balancing covariates in randomizing the last 49 subjects at the end of the trial. For subjects who received intravenously administered alteplase, 98.2% of the subjects had true random assignment at the end of the trial and there was no voting on any of the balancing covariate in dynamically allocating the last 120 subjects at the end of the trial.

**Table 3:** Treatment differences on baseline covariates by intravenously administered alteplase status at interim analysis (*N* = 316)

Baseline covariates	IV alteplase (*n* = 237)			Without IV alteplase (*n* = 78)		
	Treatment (*n* = 120)	Control (*n* = 117)	*p* value	Treatment (*n* = 45)	Control (*n* = 33)	*p* value
Age (mean, SD)	69.16 (12.32)	68.43 (14.89)	0.68	69.76 (16.31)	69.45 (13.19)	0.93
NIHSS score (mean, SD)	16.38 (5.35)	16.69 (5.75)	0.66	16.93 (5.60)	15.73 (5.43)	0.34
Sex (% female)	50.00%	51.28%	0.844	57.78%	57.58%	0.99
ASPECTS score (% (8–10))	66.67%	70.09%	0.572	60.00%	72.73%	0.24
Baseline occlusion location (% MCA M1)	73.33%	72.65%	0.906	73.33%	75.76%	0.81

*ASPECTS* Alberta Stroke Program Early CT score, *MCA M1* M1 segment of middle cerebral artery, *NIHSS* National Institute of Health Stroke Severity Scale, *SD* standard deviation, *IV* intravenously administered

## Discussion and conclusion

This study describes the implementation of minimal sufficient balance randomization, a covariate adaptive randomization strategy, in the ESCAPE trial. Our results demonstrate that overall group balance was achieved in dynamically allocating subjects to treatment arms in the ESCAPE trial both at the time of the interim analysis and at the time that the trial was stopped. This suggests that this covariate adaptive randomization strategy can assist data and safety monitoring boards to make reliable decisions about futility or efficacy in a randomized controlled trial.

Similar to all adaptive trial design approaches, the integrity of this approach relies upon the immediate availability and accuracy of data used, and, in this case, data on the covariates used to implement the randomized minimization approach. Because of the nature of stroke treatment, fast treatment decisions are necessarily taken without certainty on all preclinical data. For example, a patient’s age might be determined just upon the year of birth, but later require correcting by 1 year because the complete date of birth becomes known several hours later. We instituted a policy of correcting the data based upon the main trial database, which was considered the source of truth. This introduces a small degree of imprecision in the application of the approach since randomization will depend upon what values are in the trial randomization database. It is expected that the importance of this kind of inaccuracy will wane over time as the trial accrues more and more patients.

It was particularly important to us, in planning the ESCAPE trial, to ensure that we maintained balance on key variables at the time of interim monitoring. Because the trial was relatively small in sample size, the use of the minimal sufficient balance algorithm served as potential insurance against the possibility of random imbalance on key prognostic variables. This provided some confidence to the Data Safety and Monitoring Board that the results would not be confounded by covariate imbalance at the interim look. These results show that minimal sufficient balance randomization is able to achieve a high rate of true random allocation compared to other commonly used randomization schemes such as permuted blocks.

This use of a minimal sufficient balance algorithm in this trial demonstrates the potential advantage of this covariate adaptive design in achieving balance of key prognostic variables in a small-sampled RCT with pre-specified interim analyses thresholds. In permuted block designs, the last subject(s) in a given block size will have a deterministic treatment assignment, with the proportion with true random allocation varying by block size. In comparison, the degree of true random allocation was very high in the ESCAPE trial, preserving the fundamental nature of the RCT. Further research is needed to demonstrate how several parameters of the minimal sufficient balance randomization (e.g., such as the number and nature of baseline prognostic factors, number of anticipated interim analyses, tolerance limits, and biased coin probability) can aid the determination of the expected sample size for a trial design.

This study has limitations. The minimal sufficient balance algorithm as proposed by Zhao et al. was implemented in the ESCAPE trial by stratifying on a prognostic factor (intravenously administered alteplase) while balancing on the remaining five baseline covariates within each strata. This stratified minimal sufficient balance randomization was adopted because of variation in standard of care clinical use. Future research will use computer simulations to examine the statistical properties of this variant of minimal sufficient balance randomization scheme under a variety of data analytic conditions.

In summary, this study describes the implementation of the minimal sufficient balance, a covariate adaptive randomization scheme, in the ESCAPE trial with results from empirical data confirming balance between treatment arms across six baseline prognostic factors. The minimal sufficient balance randomization scheme is advantageous for achieving group balance in small-sampled trials. We therefore recommend minimal sufficient balance randomization for use in clinical trials where balance on multiple baseline covariates is important.

## Additional file

Additional file 1: Statistical Analysis Plan. (PDF 1202 kb)

## Abbreviations

ASPECTS: Alberta Stroke Program Early CT score
Carotid T/L: Carotid terminus with T-type or L-type occlusion
CT: Computed tomography
CTA: Computed tomography angiography
ESCAPE: Endovascular treatment for Small Core and Anterior circulation Proximal occlusion with Emphasis on minimizing CT to recanalization times trial
M1: M1 segment (or proximal segment) of the middle cerebral artery
MCA: Middle cerebral artery
NIHSS: National Institutes of Health Stroke Severity Scale score
RCT: Randomized controlled trial
