# Supplementary material for: Minimal sufficient balance randomization for sequential randomized controlled trial designs: results from the ESCAPE trial
Source: Trials. 2017 Nov 2;18:516. doi: 10.1186/s13063-017-2264-1 (PMC5667454; doi:10.1186/s13063-017-2264-1)
Supplement: Additional file 1: — Statistical Analysis Plan. (PDF 1202 kb) [file 13063_2017_2264_MOESM1_ESM.pdf]

ESCAPE trial: Statistical Analysis Plan

1. Objectives of the study

To determine if endovascular mechanical thrombectomy is superior to best standard of care in an imaging-selected population of acute ischemic stroke patients.

2. Study Design

Prospective, randomized, open-label with blinded outcome evaluation (PROBE) design.

3. Sample size

There is now empirical evidence that the mRS scale, when categories 5 and 6 are combined, is a truly interval scale meaning that the difference in utility (or function) between any one step increment in the scale is the same; thus, it is unlikely that the proportional odds assumption will be violated.[1] The trial is powered to detect a statistically significant shift in the distribution of scores on the mRS scale at 3 months between the treatment and the control arm, assuming that categories 5 and 6 (bedbound with severe disability, and death) are collapsed into one category, and the effect leads to an assumed common odds ratio of 1.8. The expected distribution of outcomes is based upon the pooled experience from the literature [SWIFT study, STAR registry, Calgary data, IMS-3 study limited to an ESCAPE population, ALIAS part 1 study limited to an ESCAPE population[2-6]] and a 5% increase in favourable outcome per mRS category. [Figure 1] In IMS-3 there was an average 2% non-significant improvement in favourable outcomes per mRS category; in SWIFT (which compared two endovascular devices), there was an average 8% improvement in favourable outcomes per mRS category.[3, 4] Using Calgary data, the common OR for improvement across the mRS scale with endovascular treatment compared to IV only was 2.1 (CT_{95} 1.1-4.2). [Hill M, unpublished data]

A total sample size of 188 patients per group is required for 90% power, two-sided alpha at 0.05 and 1:1 randomization to detect a common OR of 1.8, based upon the expected distribution shown below (figure 1). The sample size calculation is based upon Whitehead's power formula.[7] The sample size is estimated without knowing the distribution of pre-specified confounding variables. We assume no cross-overs. Allowing for losses-to-follow-up (of up to 6%, n=24/400), a total required sample size of 400 ideal patients is anticipated. However, we know that the effect size is highly dependent upon very rapid treatment times and have planned to continue enrolling patients until up to 200 endovascular patients have been treated with a CT-to-first reperfusion time < 90 minutes. Currently, in our initial patients we are meeting this quality metric in 60% of our patient population, compared to our desired 80% or greater. We conservatively assume that the effect size is absent if we do not achieve our target treatment times. Therefore,

(1) the trial is powered to detect an effect size based upon 400 ideal patients

(2) the maximum sample size for the trial will be 500 patients to ensure that 200 patients in the endovascular group meet the target treatment times (i.e. are ideal patients).

Figure 1. Projected outcomes by mRS category

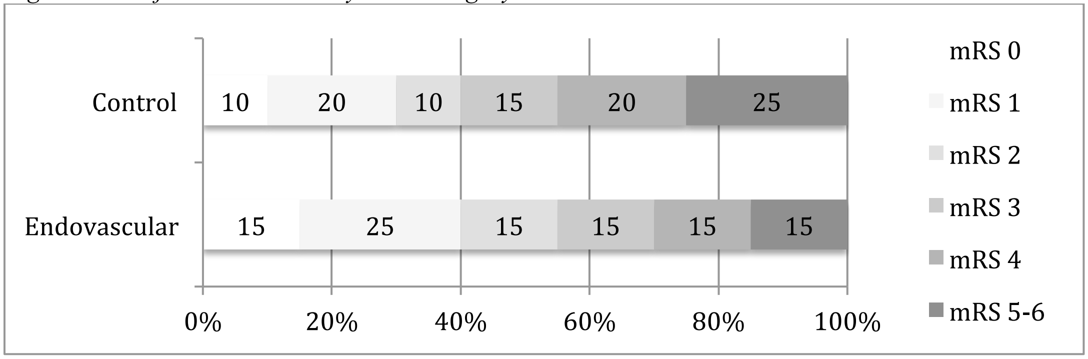

4. Interim Analyses

We will plan for two interim analyses after 125 and 300 patients have completed 3-month follow-up. At the safety analyses (n=125) the DSMB will be asked to consider safety only. The second analysis (n=300) will assess for safety and overwhelming efficacy and make recommendations accordingly. We will use O'Brien-Fleming boundaries at the interim analysis as follows:[8, 9]

There are no established techniques for the assessment of interim trial efficacy boundaries using an ordinal logistic regression model (proportional odds model). Instead, we will revert to a simple dichotomous analysis of the mRS score at 0-2 defined at 90 days from randomization. [This approach is also being used in other interventional treatment trials in stroke.] The Z-statistic for this analysis shall be derived from the normal approximation of the binomial distribution as an unadjusted two-sample test of proportions.

O'Brien Fleming Boundary for a Binary Primary Endpoint

For a RCT comparing two treatment arms with respect to a binary outcome and one interim analysis, the binary test statistic is given as

$$Z_k = \frac{(p_{Ak} - p_{Bk})}{\left\{\sqrt{\overline{p_k}(1-\overline{p_k})/\left(\frac{1}{n_A}+\frac{1}{n_B}\right)}\right\}}, k = 1, \ldots, K = 2$$

where $\bar{p}_k = \frac{p_{Ak}+p_{Bk}}{2}$, *where* P_{Ak} *and* P_{Bk} *are the estimated proportions in treatment arms A and B at stage k, respectively. The two-sided sequential test based on O'Brien & Fleming boundary is given as*

1. At stage 1 (interim analysis, n=300): Reject H_0 *and stop the trial at stage k if:*

$|Z_k| \geq C_B(2_z 0.05)\sqrt{2} = 2.834$

Else if $| Z_k | < C_B(2_z 0.05)\sqrt{2} = 2.834$, *continue to stage 2.*

2. *At stage 2 (final analysis): Reject* H_0 *at stage k if:* $| Z_k | \geq C_B(2_z 0.05) = 2.004$

Therefore, for a RCT with one interim analysis and a final analysis (i.e., K = 2), the critical boundaries at Stage 1 and Stage 2 (final analyses) are 2.834 and 2.004, respectively.

Instructions to DSMB: Stopping Rules/Guidance

- **Thus, if the Z statistic is greater than 2.834 at the interim analysis, the committee will then consider that there is statistical evidence for overwhelming efficacy.**

- **The committee should then consider the primary outcome analysis using the ordinal logistic regression (proportional odds model).**

- **Next the committee should consider any effect upon all-cause mortality**

- **Finally the committee should consider the treatment effect in two subgroups: patients treated with IV tPA and patients not treated with IV tPA.**

The committee is then entrusted with a decision to make recommendations about the continuation of the trial in the context of the data and the context of the current and known evidence about endovascular stroke treatment using their best judgment.

Because of past experience with IMS-3 and published results from the Penumbra, SWIFT and TREVO trials, we have confidence in the safety of endovascular treatment. However, as has evolved in past trials of hemicraniectomy in stroke, and in the SWIFT trial, there is a possibility that mortality in the control group may be higher than the treatment group, even when a morbidity benefit is not evident. Because our objective is to demonstrate a morbidity benefit, the DSMB will be asked to consider such a result (mortality benefit) carefully before recommending that the trial continue or be halted. This will be facilitated by having stringent futility and overwhelming efficacy boundaries using O'Brien-Fleming methods (which are known to be conservative at the interim analysis stage). There will be no analysis for futility.

5. Definition of the target population
    5.1. Efficacy population
    All patients enrolled in the trial randomized on an intent-to-treat basis.

    5.2. Safety population
    All patients enrolled in the trial who received the intervention, for the endovascular group. All patients in the control group who received best standard of care.

6. Randomization

Randomization will be managed using a custom SQL server-based database that will instantly and dynamically assign treatment using the minimal sufficient balance algorithm. Randomization will therefore be conducted over the internet via a desktop computer or a web-enabled smart phone.

Randomization will be 1:1. Allocation will be 1:1 set at p(0.5) for the first 40 patients. Thereafter, a minimization algorithm (minimal sufficient balance) will be utilized to ensure ongoing balance in the trial on the following factors:

Age
Baseline NIHSS score
Sex (male or female)
Baseline ASPECTS score, dichotomized as 6-7 or 8-10.
Intracranial occlusion location, dichotomized as MCA or ICA.
IV tPA use (yes or no)

The minimal sufficient balance (MSB) randomization is a minimization procedure that preserves balance in smaller trials, such as this one, where imbalances in important baseline prognostic variables may occur by chance and confound the primary outcome. In addition, it preserves a greater degree of randomness in patient allocation compared to permuted block designs.[10] Because of the MSB process, randomization assignments will be stochastically derived in real time using a interactive web-site and therefore concealment can never be breached. Allocation will be open.

Reliance on a process that requires real-time data entry makes the process susceptible to error. For example, incorrect information (eg. wrong sex or incorrect site of occlusion) could be mistakenly entered into the randomization process and affect the minimization algorithm. Post-hoc, when such errors become known, the quality-controlled database entry will be considered the source of truth and the randomization database will be updated to ensure that ongoing randomization utilizes the most correct data to determine balance in an ongoing way.

The randomization process is not blocked, nor stratified by site. Therefore, the number of patients enrolled into each arm of the study may not be exactly even at the time the study is completed. The proportion of patients enrolled into each arm at each site may also vary and not be equally distributed. These decisions were taken explicitly with the knowledge and belief that balance on 6 key patient characteristics in the trial overall are more important than balance by site.

7. Blinding

Treatment assignment is open-label. Blinding of the outcome assessment at 90 days will be ensured at the site by having a person who was not involved in the acute treatment period conduct the assessment.

8. Missing data

Under the ITT principle, all patients who are randomized are included in the analysis. Therefore, missing data, especially in the outcome measures, can be problematic. Thus every effort will be made to keep all missing data to a minimum. In the event that, despite the clinical centers' best efforts, there are missing data, then for the primary outcome analysis, missing outcome data will be imputed by assuming the missing mRS score at 3 months to be unfavorable. We will assign the worst possible score. If the patient is known to alive, we will impute a score of 5. If the patient is not known to be alive or dead, we will impute a score of 6.

Furthermore, if the assessment was conducted outside of the protocol-specified time window, data obtained are still included in the analysis, with the rationale that it is a more accurate measure than those obtained by imputation. At a minimum 90-day outcome assessments will be accepted within a +/- 30-day window.

For sensitivity analysis, the missing outcome values will be imputed using the following methods:

- If one-month or 5-day/discharge outcome scores are available, carry forward those values; else, assume the worst case.
- Assign failure (worst possible score) to all subjects with missing 3-month outcome data.
- Multiple imputation method based on the regression model
- Hot-deck or nearest neighbor method, using clinical site, age, sex, baseline NIHSS, baseline serum glucose, baseline ASPECTS, IV tPA use, treatment group as classification variables.
- Regression method, with age, sex, baseline NIHSS, baseline serum glucose, IV tPA vs. non-IV tPA, and treatment group as covariates.
- In the endovascular group, impute values for LTFU with failure (worst possible score), and in the control group, with success (the best-worst case imputation) in the intervention group.

Similar imputation methods will be employed for secondary categorical outcomes. For the raw NIHSS score, multiple imputation, regression, and mean substitution methods will be used in the sensitivity analyses. Missing covariate data, if any, will be imputed using either multiple imputation or regression method, if needed.

9. Efficacy Analysis

   9.1. Primary analysis

   The primary analysis will be conducted on an intention-to-treat basis using a proportional odds model (ordinal logistic regression) with an assumed common odds ratio of improvement on the mRS. We will test whether the proportional odds assumption is satisfied using chi square test. A secondary analysis will use an additive multivariable model adjusting for all the minimization variables included as co-variables. Only main effects will therefore be considered in this model. The results will be expressed as an odds ratio with 95% confidence limits. Additional analyses will include a safety population analysis defined to include only those patients who

received an interventional procedure, an on-protocol analysis including those patients who were treated according to protocol. If the proportional odds assumption is not satisfied we will revert to a binary outcome of independence (mRS 0-2) vs. dependence or death (mRS 3-6) at 90 days. This will be assessed using a Fisher's exact test.

The primary analysis will be unadjusted. Because the randomization is being balanced a priori according to key prognostic variables (age, sex, NIHSS, ASPECTS, occlusion location and tPA use), we expect that the unadjusted analysis will be similar to the adjusted analysis.

A revised statistical analysis plan may be modified according to the statistical distribution of variables and finalized prior to breaking the blind.

9.2. Secondary analyses

Pre-specified secondary outcome and safety analyses of proportions will be conducted in a similar way to the primary analysis using logistic regression or using a multivariable generalized linear model with log link to derive risk ratios directly. Pre-specified secondary analyses will include the following:

- 9.2.1. Adjusted analysis on the primary outcome using key stratifying variables from the MSB algorithm (age, sex, tPA use, ASPECTS score, NIHSS score, occlusion location)
- 9.2.2. Adjusted analysis using other known predictors of outcome after stroke (6 variables above, time, serum glucose, atrial fibrillation, ipsilateral carotid artery stenosis/occlusion, diabetes mellitus, blood pressure at baseline, miFUNCTION score)
- 9.2.3. Dichotomized outcome on the 90-day: mRS (independent 0-2 vs. dependent 3-6), the NIHSS 0-2 vs. 2-42, Barthel index 95-100 vs. 0-90, mortality. Each dichotomized outcome will be modeled as a function of key covariates (as per 8.2.2) using logistic regression model. The odds ratio and the corresponding 95% confidence interval for each covariate will be reported
- 9.2.4. Reperfusion and recanalization analysis based upon angiographic data (endovascular group) and CTA data (control group). Proportion of patients with early recanalization, extent of recanalization.
- 9.2.5. Analysis of the role that time from onset and time from CT imaging plays in predicting outcome defined by the mRS by group.
- 9.2.6. Analysis on the primary outcome using baseline imaging as a stratifying variable (ASPECTS 8-10 vs. 5-7, multi-phase CTA collaterals good vs. intermediate and perfusion CT small vs. intermediate infarct core) Simple effects and interactions will be considered in this analysis.
- 9.2.7. Analysis of quality of life, measured using the EuroQOL. We will report the unadjusted distribution by group.
- 9.2.8. Economic analysis will be conducted using Canadian hospital data and quality of life measure to estimate treatment utility.

9.3. Exploratory analyses

Any other analyses, that arise after the trial SAP is finalized, will be considered exploratory and stated as such. No adjustments for multiplicity will be made for these exploratory analyses.

10. Safety Analysis

10.1.1. Serious adverse event reporting. Proportions of adverse events by type.

10.1.1.1. Adjusted analysis of predictors of symptomatic ICH by group

10.1.1.2. Device-related complications and adverse events listing

References

1. Hong KS, Saver JL. Years of disability-adjusted life gained as a result of thrombolytic therapy for acute ischemic stroke. *Stroke; a journal of cerebral circulation*. 2010;41:471-477
2. Pereira VM, Gralla J, Davalos A, Bonafe A, Castano C, Chapot R, et al. Prospective, multicenter, single-arm study of mechanical thrombectomy using solitaire flow restoration in acute ischemic stroke. *Stroke*. 2013;44:2802-2807
3. Saver JL, Jahan R, Levy EI, Jovin TG, Baxter B, Nogueira RG, et al. Solitaire flow restoration device versus the merci retriever in patients with acute ischaemic stroke (swift): A randomised, parallel-group, non-inferiority trial. *Lancet*. 2012;380:1241-1249
4. Broderick JP, Palesch YY, Demchuk AM, Yeatts SD, Khatri P, Hill MD, et al. Endovascular therapy after intravenous t-pa versus t-pa alone for stroke. *N Engl J Med*. 2013;368:893-903
5. Bhatia R, Shobha N, Menon BK, Bal SP, Kochar P, Palumbo V, et al. Combined full dose iv and endovascular thrombolysis in acute ischemic stroke. *Int J Stroke*. 2012;In press
6. Hill MD, Martin RH, Palesch YY, Tamariz D, Waldman BD, Ryckborst KJ, et al. The albumin in acute stroke part 1 trial: An exploratory efficacy analysis. *Stroke*. 2011;42:1621-1625
7. Whitehead J. Sample size calculations for ordered categorical data. *Stat Med*. 1993;12:2257-2271
8. De Mets DL, Furberg CD, Friedman LM. *Data monitoring in clinical trials*. New York: Springer; 2006.
9. Jennison C, Turnbull BW. *Group sequential methods with applications to clinical trials*. New York: Chapman & Hall; 2000.
10. Zhao W, Hill MD, Palesch YY. Minimal sufficient balance – a new strategy to balance baseline covariates and preserve randomness of treatment allocation. *Stat Meth Med Res*. 2012;In press
